# Improvement of CZTSSe film quality and superstrate solar cell performance through optimized post-deposition annealing

**DOI:** 10.1038/s41598-022-20670-1

**Published:** 2022-09-28

**Authors:** V. Pakštas, G. Grincienė, A. Selskis, S. Balakauskas, M. Talaikis, L. Bruc, N. Curmei, G. Niaura, M. Franckevičius

**Affiliations:** 1 Center for Physical Sciences and Technology, Sauletekio Av. 3, 10257 Vilnius, Lithuania; 2 Institute of Biochemistry, Life Sciences Center, Vilnius University, Sauletekio 7, 10257 Vilnius, Lithuania; 3 Institute of Applied Physics, 5 Academiei Str., Chişinău, 2028 Moldova

**Keywords:** Materials science, Materials for devices

## Abstract

Improving the performance of kesterite solar cells requires high-quality, defect-free CZTS(Se) films with a reduced number of secondary phases and impurities. Post-annealing of the CZTS films at high temperatures in a sulfur or selenium atmosphere is commonly used to improve the quality of the absorbing material. However, annealing at high-temperatures can promote material decomposition, mainly due to the loss of volatile elements such as tin or sulfur. In this work, we investigate how the additional step of sulfurization at reduced temperatures affects the quality and performance of CZTSSe based solar cells. A comprehensive structural analysis using conventional and high resolution XRD as well as Raman spectroscopy revealed that the highest CZTSSe material quality with the lowest structural disorder and defect densities was obtained from the CZTS films pre-sulfurized at 420 °C. Furthermore, we demonstrate the possibility of using Sb_2_Se_3_ as a buffer layer in the superstrate configuration of CZTSSe solar cells, which is possible alternative to replace commonly employed toxic CdS as a buffer layer. We show that the additional low-temperature selenization process and the successful use of Sb_2_Se_3_ as a buffer layer could improve the performance of CZTSSe-based solar cells by up to 3.48%, with an average efficiency of 3.1%.

## Introduction

The increasing demand for energy, which leads to a rapid consumption of fossil fuels, arouses interest in alternative and renewable energy sources. Among most prominent renewable energies such as wind power, geothermal energy, biomass, etc., solar energy is considered the cleanest and most abundant energy resource. Although silicon is currently the most commonly used semiconductor in photovoltaic modules, other semiconductor materials that convert the energy of sunlight into electricity have also been widely attempted in solar cells as well^1,2^. The kesterite structure quaternary compounds Cu_2_ZnSnS_4_ (CZTS), Cu_2_ZnSnSe_4_ (CZTSe) or Cu_2_ZnSn(SSe)_4_ (CZTSSe) are considered emerging and potentially promising semiconductors for thin-film photovoltaic (PV)^3^. They consist of environmentally friendly Earth abundant elements, have high absorption coefficients of 10^−4^–10^−5^ cm^−1^ and high hole concentration covering a range from 10^15^ to 10^20^ cm^−3^ ^4,5^. The record PCE of kesterite-based solar cells currently is only 12.6%, which obviously still too low to be of commercial value^6^. The low performance is mainly associated with the crystallization process which is difficult to control^7^ and leads to the elemental disorder and a high density of bulk and surface defects^8^.

Most kesterite-based solar cells have a regular architecture with molybdenum-coated glass (Mo) as the back contact and CdS as the buffer layer (CdS, ZnS), both of which are directly contacted with kesterite. At high temperatures, the Mo-CZTS interface is susceptible to degradation, leading to the formation of secondary phases such as Cu_2_S, ZnS and SnS and the growth of MoS_2_, which significantly increases the defect density and impairs open-circuit voltage and fill factor values^9,10^. These disadvantages can be partially avoided by using superstrate configuration devices where molybdenum is no longer indispensable. In such configuration, the kesterite is deposited between the transparent conductive layer and the back contact, which serves as hole transporting layer. One of the simplest and most versatile methods for producing CZTS kesterite absorber materials is the spray pyrolysis technique^11^. It requires low production costs and allows easy control of the thin film parameters by choosing the optimal concentrations of the molecular precursor for CZTS, the coating rate and the substrate temperature. However, the efficiency of CZTSSe based solar cells processed by spray pyrolysis technique is still low and is comparable to the efficiencies of devices processed using other wet chemistry methods^12^. Expectedly, that among the many factors that limit PCE, the most important in spray pyrolysis deposited films are defects at the grain boundaries where high density of traps could be formed. These traps can be responsible for the formation of the additional electronic states or even intra-bandgap states^13^ leading to a limited charge carrier separation and increased recombination in the material, therefore disrupting charge carrier motion in the semiconductor and consequently lowering performance parameters of the solar cell.

The development of high-performance kesterite solar cells requires high-quality, defect-free CZTS(Se) films with a reduced number of secondary phases and impurities. To achieve this, it is essential to precisely control the quality of the functional layers constituting the device and, in particular, each CZTS film formation step. The as-deposited CZTS films deposited by spray pyrolysis are usually of poor quality with lots of defects and low crystallinity or even solvent residues in the film^14–16^. An important step to improve the material quality is the high-temperature post-annealing of the pre-deposited CZTS absorbing material, either in sulfur or selenium atmosphere, to obtain CZTS or CZTSSe films^17^. Post-annealing is usually performed at high temperatures of 500 °C or more, resulting in decomposition of the CZTS film due to loss of SnS and S from the sample^18^. To prevent loss of sulfur and to obtain stoichiometric CZTS absorber material, annealing is usually performed in a sulfur vapor atmosphere^19^. While for CZTSSe absorber material, the pre-deposited CZTS films are selenized to replace the sulfur with the appropriate selenium content. On the other hand, the combined two step annealing process has never been used for the preparation of solution processed CZTSSe absorber material.

In this work, we performed low-temperature sulfurization of the spray pyrolysis deposited CZTS films prior to their selenization. The spray pyrolysis deposited CZTS films were annealed at different temperatures in a sulfur atmosphere. We investigated the effect of pre-sulfurization on the quality of CZTSSe absorber materials and demonstrated the benefit of the additional sulfurization process on superstrate solar cell performance. Furthermore, we have demonstrated the possibility of using Sb_2_Se_3_ as a buffer layer in the superstrate configuration of CZTSSe solar cells. Through the successful use of antimony selenite and an optimized selenization process, we were able to improve the quality of the CZTSSe absorber material and increase solar cell performance of the following device architecture: FTO/TiO_2_/Sb_2_Se_3_/Cu_2_ZnSnSe_3.2_S_0.8_/PEDOT:PSS/Au up to 3.48%. Based on recent literature reports, we believe this is one of the highest values for the Cd-free superstrate CZTSSe solar cell processed by spray pyrolysis technique^12^.

## Materials and methods

### Materials and device fabrication

**Figure 1:**
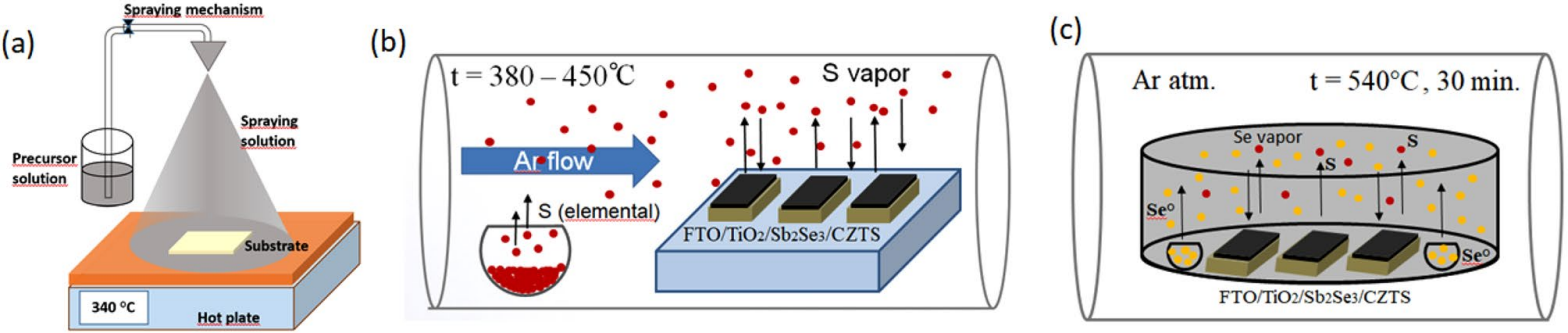
Experimental procedure for obtaining CZTSSe films. Schematic representation of the preparation of CZTS films by spray pyrolysis (**a**), followed by sulfurization (**b**) and subsequent formation of CZTSSe films by selenization (**c**) processes.

The film and device preparation involves slightly modified protocols reported previously^20^. Briefly, a thin TiO_2_ layer (thickness ~ 30–40 nm) was deposited on a transparent FTO glass substrate by spray pyrolysis. On top of this layer, thin Sb_2_Se_3_ films with a thickness of about 10—15 nm were formed by the vapour transport deposition (VTD) method^21^. Afterwards, the CZTS films were deposited by spray pyrolysis at a temperature of 340 °C. The formed film was heated to 340 °C for 0.5 h. The CZTS films were then annealed in a sulphur atmosphere at 380, 420 and 450 °C for 0.5 h. The schematic of the spray pyrolysis experiment and the sulphurisation process of the CZTS films are shown in Fig. 1a,b, respectively. The CZTS films were then selenized in a graphite box at 540 °C for 20 min (Fig. 1c)^22,23^. Subsequently, PEDOT:PSS was deposited as a hole-selective layer on CZTSSe by spin coating and the devices were finished by thermally evaporating a 60 nm thick gold layer.

### Morphology, structure and device characterization

The surface morphology of the films and the cross-section of the devices were measured with the scanning electron microscope (SEM) Helios Nanolab 650. The X-ray diffractometer SmartLab (Rigaku) was used to determine the phase and structural properties of the films. The very accurate XRD measurements were performed with the CALSA analyser on a diffracted beam, which allows the registration of XRD patterns in Cu Ka1 radiation (λ = 1.5405929 Å). The size of the crystallites was calculated using the graphical Halder-Wagner method^24,25^ implemented in the PDXL software.

Raman measurements at 785 nm wavelength excitation were performed by using RamanFlex 400 (PerkinElmer, Inc.) spectrometer. Laser radiation was restricted to 20 mW and focused on a 200-μm diameter spot size on the sample surface. The 532-nm excitation Raman measurements were performed by using Raman microscope LabRam HR800 (Horiba Jobin Yvon) equipped with the 1800 lines/mm grating and thermoelectrically cooled (− 90 ◦C) CCD camera (DU920P-BR-DD). Laser light was restricted to 2.2 mW and focused on a sample by 50 × /0.50 NA long working distance (LWD) objective. The 532-nm excitation Raman measurements of Sb2Se3 buffer layer were performed by inVia Raman microscope (Renishaw, Wotton-under Edge, UK) equipped confocal Leica microscope. Laser radiation was set to 2.2 mW and focused on a sample by using 50 × /0.50 NA long working distance (LWD) lens.

The current–voltage properties of the CZTSSe solar cells were performed with a xenon lamp light source (Oriel, model 9119, Newport) producing standard AM1.5G illumination. While changing the external bias voltage applied to the cell from –0.5 to 0.5 V we recorded the generated photocurrent with a Keithley (model 2420) digital source meter.

## Results and discussion

We have previously established that CdS layer improves CZTS adhesion to the TiO_2_ surface, which is rather poor when kesterite is directly deposited on TiO_2_^26^. However, at high temperatures, above 450 °C, the CdS no longer acts as a buffer layer because it decomposes into Cd and S ions, which then diffuse into the kesterite absorber layer preventing antisite defect formation due to partial substitution of Zn with Cd. However, Cd is toxic metal and its use in solar cell devices is a huge drawback for the environment and public health. Therefore, to avoid environmentally harmful Cd ions, here we employed thin (about 15–20 nm) Sb_2_Se_3_ layer as a buffer layer instead of CdS, which also we expect able to improve the CZTS adhesion on the surface. Moreover, the inevitable loss of Sn ions at high temperatures can be possibly substituted by Sb due to their isovalent nature^27^, being another important reason for using Sb_2_Se_3_ interlayer. The sample composition we used consists of CZTS absorbing layer deposited on top of TiO_2_/Sb_2_Se_3_ coated FTO glass substrate following spray pyrolysis technique.

The composition of the pre-deposited CZTS film and the CZTS films sulfurized at low temperatures of 380, 420, and 450 °C (hereafter referred to as pre-sulfurized) are presented in Table 1. As shown by EDX analysis, all samples exhibit a non-stoichiometric distribution of Cu and Zn atoms in the CZTS structure, containing a small amount of copper (Cu/(Zn + Sn) = 0.82–0.87) and an excess of Zn (Zn/Sn = 1.04–1.14). This non-stoichiometric distribution has been shown to promote the formation of secondary phases, defects and vacancies in CZTS films^28,29^; on the other hand it is beneficial for enhanced charge carrier transport and solar cell performance^30,31^. In addition, a certain amount of chlorine is also present in the CZTS material (see Table S1). Chlorine can also promote the formation of defects and secondary phases in CZTS films and therefore should be avoided. We observed that the chlorine content decreases from 1.88 for the as-deposited films to 0.08 for the films sulfurized at 450 °C. Consequently, presulfurization at low temperature allows maintaining the Cu and Zn-rich CZTS composition and reducing the chlorine content. Further, we provide comprehensive analysis of the presulfurization temperature effect on the material quality.

**Table 1:** As-deposited and sulfurized at different temperature CZTS components chemical ratio.

Sample	Cu/(Zn + Sn)	Zn/Sn	Cu/Sn	(Cu + Zn + Sn)/S
As-deposited	0.87	1.04	1.77	0.97
380 °C	0.85	1.08	1.76	0.99
420 °C	0.85	1.14	1.81	0.99
450 °C	0.82	1.10	1.72	0.98

**Figure 2:**
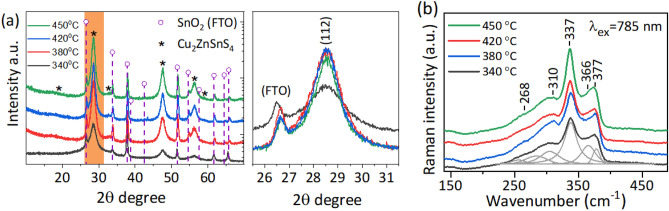
Characterization of the as deposited and sulfurized CZTS films using XRD and Raman spectroscopy. XRD pattern of CZTS absorbers: as-deposited (340 °C) and after sulfurization at temperatures of 380, 420 and 450 °C. The characteristic diffraction peaks of CZTS phase are marked with the circle, while the asterix marks the XRD patterns corresponding to FTO substrate (SnO_2_ (ICDD # 04-003-0974)) (Fig. 2a). Raman spectra of the as-deposited CZTS films (340 °C) and CZTS films sulfurized at various temperatures of 380, 420 and 450 °C in the spectral range of 150–470 cm^−1^ (Fig. 2b).

Figure 2a shows the XRD profiles of the as-deposited and pre-sulfurized CZTS thin films. The diffraction patterns of all the films confirm the formation of the tetragonal kesterite phase of CZTS (ICDD file #01-080-8225) (Fig. 2a left). The size of the crystallite domains is an important parameter determining the quality of the absorbing material and can be estimated from the XRD profiles (Fig. 2a right). The size of the crystallites calculated using the Halder-Wagner Method method described in Ref.^25^ are presented in the Table 2. The XRD peaks of the CZTS film are quite broad suggesting that the size of crystallites composing the film are small (about 2.6 ± 0.4 nm). Presulfurization of the CZTS films at low temperatures resulted in a significant increase in the size of the crystallites. The largest crystallites were obtained for the CZTS films that were pre-sulfurized at 420 °C and reached a size of about 5.6 ± 0.3 nm. Compared to unsulfurized CZTS films the crystal size increased more than two times. Instead of the tetragonal kesterite phase, no other phases were detected in the XRD patterns. However, during the crystallization of CZTS, other secondary phases such as ZnS or Cu_2_SnS_3_ may also form^32^, which crystal structures may overlap the tetragonal kesterite structure and therefore could be difficult to distinguish by X-ray diffraction method.

**Table 2:** The size of CZTS crystallites for as-deposited absorber and after the sulfurization at temperatures of 380, 420, and 450 °C, and also of CZTSSe crystallites after the selenization of above mentioned CZTS absorbers at temperature of 540 °C for 30 min.

Temperature	As-deposited	Sulfurized at temperature		
	340 °C	380 °C	420 °C	450 °C
CZTS	2.6 ± 0.4	4.0 ± 0.2	5.6 ± 0.3	4.6 ± 0.4
CZTSSe	46.2 ± 2.2	51.3 ± 1.1	54.5 ± 1.6	42.0 ± 2.5

To distinguish between the primary and secondary phases and to identify the extent of disorder in the crystalline structure of the CZTS films, we further evaluated the material quality by measuring the Raman spectra of the CZTS films (Fig. 2b). The characteristic Cu_2_ZnSnS_4_ Raman band at 337 cm^−1^ is assigned to *A* symmetry mode that involves pure vibration from sulfur^33–36^. This band is dominant in every annealing-temperature dependent spectrum shown in Fig. 2b. The less pronounced bands near 268, 293, 310, 366, and 377 cm^−1^ were found by decomposing the experimental spectral contour of 450 °C-annealed CZTS into the Gaussian–Lorentzian form components Fig. S1 (see Supplementary Information). The band near 293 cm^−1^ is attributed to a second *A* symmetry vibrations that also involves sulfur atom motion^34^. Annealing affects CZTS phase crystallinity and this change can be monitored by comparing the full width at half maximum (FWHM) of the dominant Cu_2_ZnSnS_4_ band at 337 cm^−1^. The increase of annealing temperature from 340 to 420 °C results in the narrowing of the band from 23.6 to 18.7 cm^−1^. However, the following temperature increase to 450 °C leads to broadening to 22.0 cm^−1^. This clearly shows that at 420 °C the highest degree of crystallinity is attained. The bands near 292 and 366 cm^−1^ resolved by fitting the experimental contour indicates some contribution to the spectrum from Cu_2_SnS_3_ phase^37^. The relative intensity of the 366 cm^−1^ band expressed as I_337_/I_366_ decreases at higher annealing temperature (Table 3). Previously, it was suggested that for a near-infrared excitation (785 nm), the intensity ratios Q (I_288_/I_304_) and Q ‘ (I_337_/(I_366_ + I_378_)) can be explored as a sensitive measure for Cu/Zn disorder in CZTS samples^38–40^. The relative increase in the magnitude of Q and Q’ quality factors indicate increasing order in the CZTS films. Despite the complex spectral pattern and overlapped bands, the annealing temperature-dependent alteration of the Q and Q ‘ ratios was found to change in agreement with the FWHM of *A* mode at 337 cm^−1^. The highest Q and Q ‘ ratios and thus the lowest disorder were found for 420 and 450 °C samples, respectively (Table 3).

**Table 3:** The position of the dominant *A* symmetry band, its full-width at half maximum (FWHM) values, I_337_/ I_365_, Q, and Q’ ratios for 785 nm-excited CZTS and quantity factor expressed as S/(S + Se) for 532 nm-excited CZTSSe Raman spectra.

Temperature, ^o^C	CZTS 785 nm					CZTSSe 532 nm
	ν_337_, cm^−1^	FWHM_337_, cm^−1^	I_337_/I_366_	Q	Q'	S/(S + Se)
340 (as-deposited)	337.9	23.6	2.23	0.58	1.75	0.12
380	338.4	25.3	2.20	0.70	1.82	0.15
420	336.7	18.7	2.77	0.83	2.08	0.18
450	337.1	22.0	2.76	0.71	2.38	0.18

**Figure 3:**
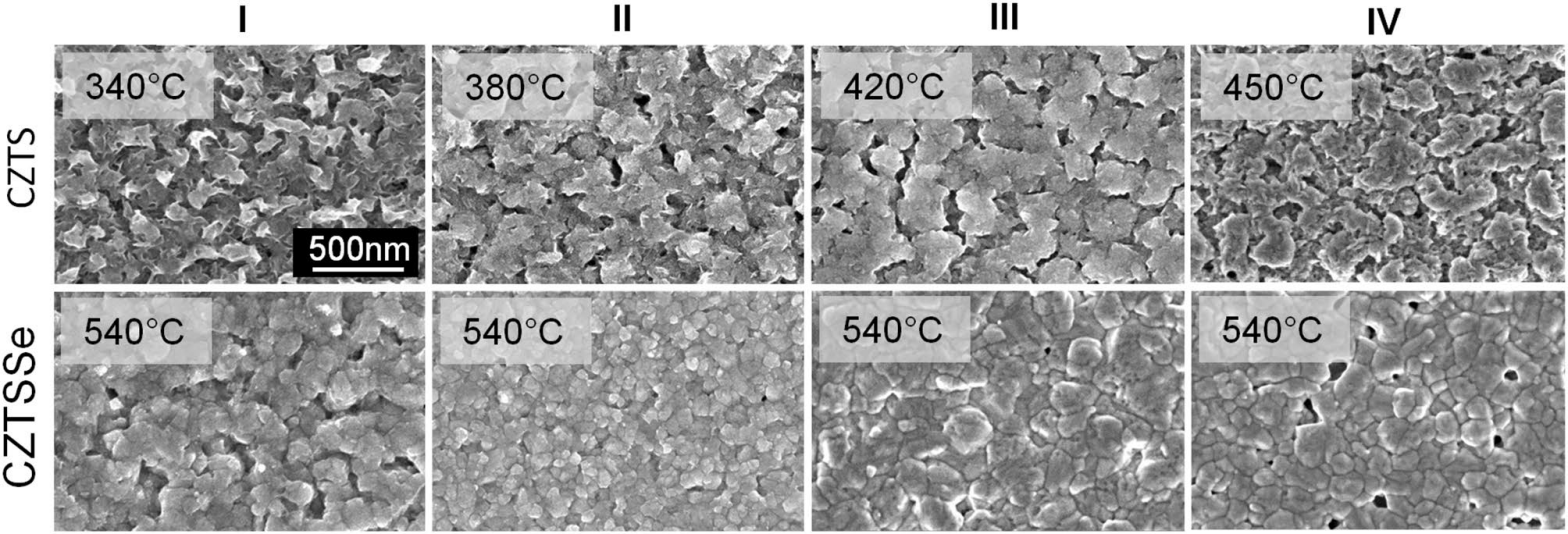
Surface morphology of CZTS and CZTSSe films. Surface morphology SEM images of the as-deposited CZTS film at 340 °C and CZTS films sulfurized at different temperatures (380, 420 and 450 °C) (top view). SEM images of CZTSSe films obtained by annealing each CZTS film at 540 °C in selenium atmosphere.

The top view surface morphology SEM images of the as-deposited CZTS films and the CZTS films sulfurized at different temperatures are shown at the bottom of Fig. 3. The as-deposited CZTS films have a porous surface with many grain boundaries and pinholes, which is consistent with our previous results^20,26^. The quality of the films slightly improves when the samples are sulfurized at higher temperatures. The most significant improvement is obtained for the films sulfurized at 420 °C, which leads to a larger grain size and a smoother film surface. This is also consistent with the results of the XRD and Raman measurements, which showed that the largest crystallites and the highest degree of crystallinity are found in the films annealed at 420 °C. Remarkably, the film morphology deteriorates with further increasing temperature, which is probably due to a possible loss of Sn and S ions leading to decomposition of the CZTS kesterite structure^41,42^.

All pre-deposited CZTS films, regardless their fabrication conditions, were further selenized at a constant temperature of 540 °C to obtain CZTSSe absorbers with the appropriate band gap. The advantage of using CZTSSe is its improved crystallinity and lower density of sulfur defects compared to pure CZTS absorber material. The surface morphology SEM images of the obtained CZTSSe films are shown at the bottom of the Fig. 3. We divided them into series according to the pretreatment conditions of the films. Each series corresponds of the CZTS films prepared at different annealing temperatures following sulfurization or without it, which were then selenized at 540 °C. It is obvious that the quality of the CZTSSe films depends on the initial conditions under which the CZTS films were formed. The CZTSSe films prepared from CZTS layers annealed at low temperatures (**I** and **II** series) consist of small crystallites with rough surface. In contrast, annealing the CZTS films at higher temperatures (420 and 450 °C) results in a smoother surface and a larger size of crystallites in the CZTSSe films (**III** and **IV** series). The best film quality is achieved in those CZTSSe films that were obtained from CZTS films selenized at 420 °C (**III** series). Overall, the selenization significantly improves the quality of all CZTSSe films, resulting in lower pinhole density, reduced grain boundaries and larger crystallites when compared to CZTS films as shown in Fig. 3, and this is particularly important when solar cells are fabricated.

**Figure 4:**
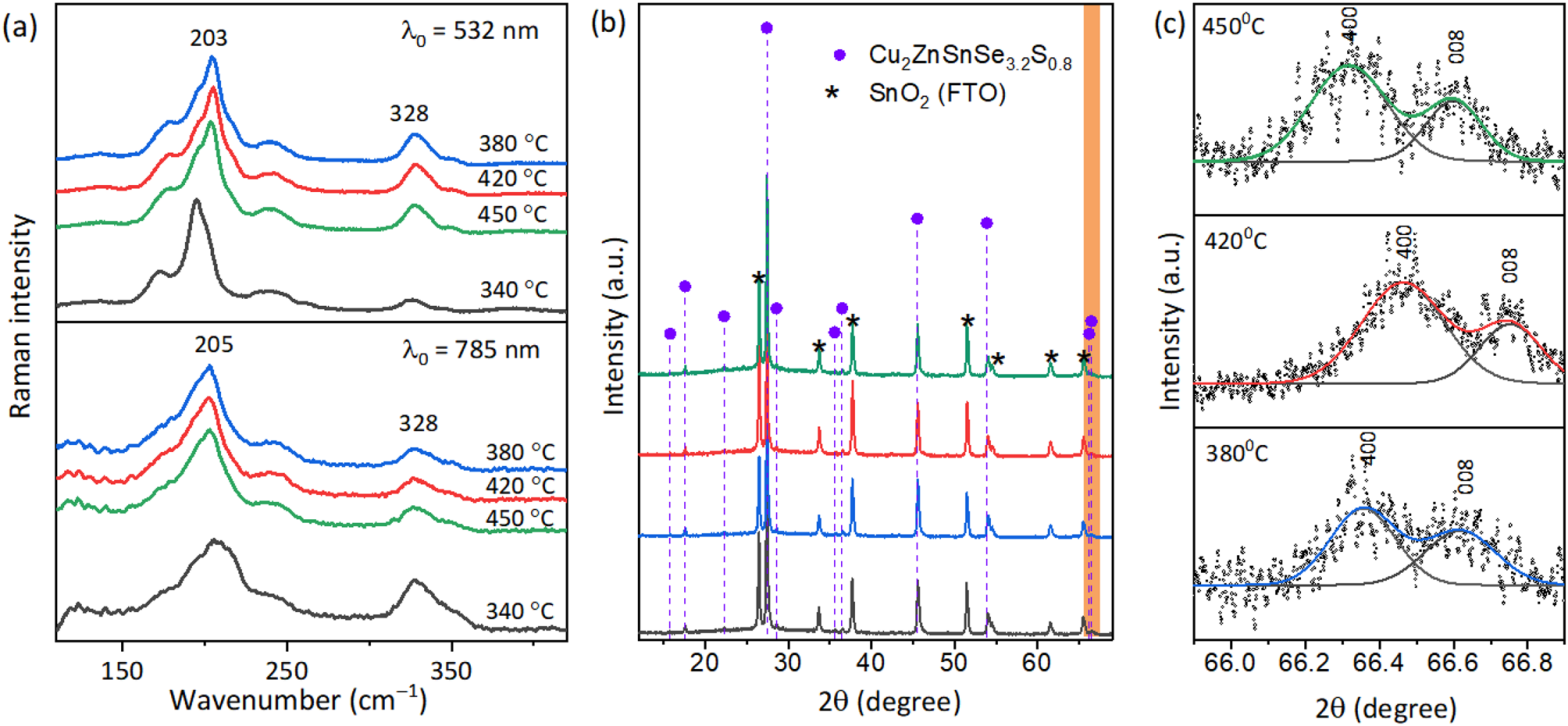
Material quality of CZTSSe films. (**a**) Raman spectra of CZTSSe as-deposited and later annealed at different temperatures in sulfur atmosphere and finally with selenium. The spectral range of 110–400 cm-1 was fitted using Gaussian–Lorentzian shape components. (**b**) the XRD patterns of CZTSSe films obtained after the selenization at temperature of 540 °C of the as-deposited CZTS films and CZTS films sulfurized at temperatures of 380, 420, 450 °C. (**c**) The fragments of XRD patterns measured with Cu Kα1 radiation representing peaks 400 and 008 of CZTSSe after the selenization at temperature of 540 °C of the CZTS films sulfurized at temperatures of 380, 420 and 450 °C.

We additionally evaluate the quality of CZTSSe absorbers obtained from CZTS films annealed at different temperatures using Raman spectroscopy, which provides insight into the selenization process^20,26,43^. Fig. 4a compares the temperature-dependent Raman spectra obtained at excitation wavelengths of 532 and 785 nm. One can observe the appearance of intense lower frequency features in the spectral range of 160 − 250 cm^−1^ which are due to the vibrations of selenium^43^. Spectra obtained with an excitation of 785 nm are more sensitive to disorder of the compound studied^39^. The CZTSSe film of the **I** series exhibits considerably broadened bands (Fig. 4 bottom). The broadening of the spectral modes results from the increased defect density, the reduced crystallinity and the changes in the local composition of the absorber. The annealing significantly reduces the FWHM values of the bands. The spectra obtained with wavelengths of 532 and 785 nm offer the possibility to evaluate the S/(S + Se) ratio^20,43^. The dominant vibrations of the Se, S and Se + S anions in CZTS are expected to occur in the frequency ranges 170–205, 280–400 and 205–280 cm^−1^. Such separation can be used to determine the elemental ratio of S and Se anions:

$$\frac{S}{S+Se}=k\frac{{A}_{s}}{{A}_{S,Se}+ {A}_{S}}+C$$

where k = 1.26(3), C = −0.046(17), AS and AS,Se are integral areas in the spectral ranges 270–380 and 150–260 cm^−1^, respectively. The values indicating the sulphur content in the CZTSSe films, S/(S + Se), are given in Table 3. Annealing in selenium atmosphere at 420 °C and above clearly increases the amount of sulfur anions in the crystal lattice.

Figure S2 compares 532-nm excited Raman spectra obtained from the top (CZTS) and bottom (glass) sides of the as-deposited CZTS sample. The bottom glass side exposes the 15–20 nm thick Sb_2_Se_3_ layer. No Sb_2_Se_3_ Raman bands were detected in the spectrum of the top side. The dominant band at 336 cm^−1^ and the shoulders at 296 and 374 cm^−1^ are assigned to CZTS vibrations. In contrast, the spectrum of the bottom contains spectral modes directly associated with the Sb2Se3 layer (Fig. S2). The modes at 152, 191 and 211 cm^−1^ are assigned to the B_3g_, A_g_, and B_1g_ symmetry vibrations of the Sb_2_Se_3_. No spectral bands were found near 250 cm^−1^, indicating the absence of oxidised antimony, Sb_2_O_3_^44^. Taken together, the Raman data indicate an intact Sb2Se3 buffer layer for the sample that was not previously subjected to the selenisation step.

Figure 4b shows the XRD patterns of the CZTSSe films obtained from the as-deposited and the pre-sulfurized CZTS films. The first insight shows that all CZTSSe films have identical XRD diffraction patterns, regardless of the pre-treatment conditions of the CZTS films. The representative XRD diffractograms show the formation of a phase-pure tetragonal kesterite Cu_2_ZnSn(S,Se)_4_ (ICDD # 04-019-1848) structure and additional SnO_2_ (ICDD # 04–003-0974) lines originating from the FTO substrate. Based on previous reports of lattice spacing as a function of kesterite composition^20,45^, we can accurately determine the kesterite phase being Cu_2_ZnSnSe_3.2_S_0.8_. As expected, selenization of CZTS films leads to a tenfold increase in crystallite sizes, with the largest crystallites obtained for the **II** series films (pre-sulfurized at 420 °C), as shown in Table 2.

We have previously shown that the distance between kesterite (CZTS) peaks 200 and 004 allows an assessment of quantity of Cu_Zn_ point defects or the degree of disorder in the positioning of Cu and Zn ions in the 2d plane of the tetragonal crystal lattice of CZTS kesterite^46^. A larger distance between the peaks 200 and 004 corresponds to a lower degree of disorder. However, due to the low spatial resolution of the conventional XRD system, the distance between the two peaks is difficult to resolve. To overcome this limitation, XRD with embedded CALSA analyzer is used to distinguish between two diffraction peaks that are close to each other. Alternatively, here we used this method to evaluate the disorder in the CZTSSe kesterite films. Since the intensity of CZTSSe kesterite peaks 200 and 004 is much lower compared to the CZTS kesterite, we deliberately chose to analyze the peaks at 400 and 008 of CZTSSe, being located at a 2θ angle of about 66.5 degrees, because the difference between the intensity maxima of these peaks gives information about the degree of order/disorder in the CZTSSe material. Figure 4c shows abovementioned peaks of CZTSSe layers, which before the selenization were sulfurized at temperatures of 380, 420, and 450 °C, respectively. It is noteworthy that peaks 400 and 008 of the as-deposited sample and the subsequently selenized sample are not shown because the intensity is too low and the peaks could not be resolved. Table 4 shows the 2θ values of peaks 400 and 008 and the distance between them in degrees. The largest spacing and consequently the highest degree of disorder was obtained for the CZTSSe film sulfurized at a temperature of 380 °C, while the lowest degree of disorder was for the sample sulfurized at a temperature of 420 °C. This is an important result demonstrating the possibility to accurately estimate the information about point defects in the CZTSSe material.

**Table 4:** 2 angles of CZTSSe peaks 400 and 008 and distance between those peaks, 2_008_ − 2_400_ for CZTSSe films sulfurized at different temperatures prior to the selenization.

Sulfurization temperature, °C	2_400_, deg	2_008_, deg	2_008_–2_400_, deg	FWHM	
				2_400_	2_008_
As-deposited	n.d	n.d	–		
380	66.357	66.618	0.261	0.2071 ± 0.0098	0.2271 ± 0.0151
420	66.464	66.752	0.288	0.1930 ± 0.0072	0.1754 ± 0.0075
450	66.319	66.601	0.282	0.2429 ± 0.0087	0.1788 ± 0.0105

**Figure 5:**
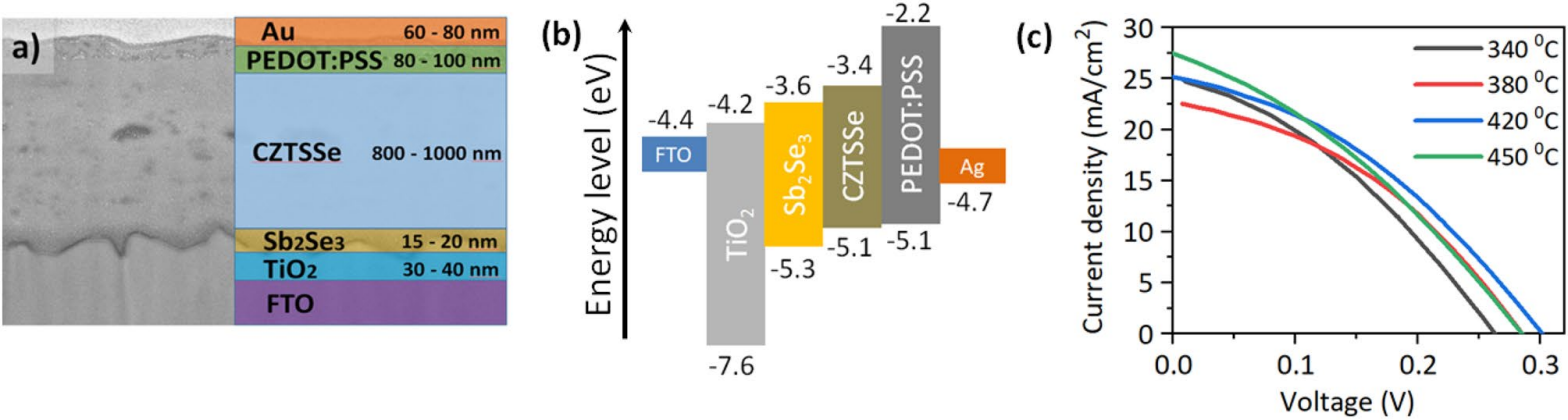
Structure and characteristics of CZTSSe solar cells. FIB-made cross-section SEM (left) and schematic CZTSSe solar device structure (right) (**a**). Energy level diagram of the materials used in CZTSSe solar cell device fabrication (**b**). Current–voltage curves of the CZTSSe devices prepared from CZTS films annealed at different temperatures (**c**).

To determine how the pretreatment conditions of the CZTS films affect the optoelectronic properties of the CZTSSe absorber materials, we investigated the photovoltaic properties of the conventional superstrate CZTSSe solar cells. The structure and cross-section SEM of the investigated CZTSSe solar cells and the energy level diagrams of the used materials are presented in Fig. 5a,b. The current- voltage (I-V) characteristics of the CZTSSe devices that showed the best performance are shown in Fig. 5c and their photovoltaic parameters are summarised in Table 5. The best performing devices were obtained with the films that were sulfurised at a temperature of 420 °C and showed a power conversion efficiency (PCE) of 3.48%. They also showed the highest values for open circuit voltage (Voc = 300 mV) and fill factor (FF = 39.4%) among all other devices. The lowest power conversion efficiencies were obtained for the CZTSSe solar cells, which were made from CZTS films that were not subjected to sulfurization or those sulfurized at an elevated temperature of 450 °C. The statistics of the photovoltaic parameters of the devices shown in Fig. S3 reflects a clear trend between the device performance and the pre-treatment conditions of the CZTS films. It is evident that the increase in PCE is mainly due to the improved device performance parameters such as fill factor and open circuit voltage. These improvements also agree well with the results of the structural analysis, where the lowest degree of structural disorder and improved crystallinity were found for the films sulfurized at 420 °C. At this point, we should emphasise that while sulphurization of CZTS films is an inevitable process to obtain CZTSSe solar cells with decent performance, even a small variation in annealing conditions can greatly affect the quality of CZTSSe films and the final performance parameters of the solar cell devices. The use of the Sb_2_Se_3_ buffer layer is another important aspect that can be considered in the fabrication of solar cells. It is obvious that the photovoltaic parameters of CZTSSe solar cells depend not only on the processing conditions, but also on the architecture of the device. The distribution of photovoltaic parameters in CZTSSe solar cells with an Sb_2_Se_3_ buffer layer has a lower standard deviation variance than in previously fabricated solar cells with a CdS buffer layer^20,26^. This could be attributed to the more favorable alignment of the energy levels of Sb_2_Se_3_ with CZTSSe and its more stable nature compared to the CdS layer. Here we have additionally measured incident photon to electron conversion efficiency (IPCE) of CZTSSe solar cells which is presented in Fig. S4. The overall photon to electron conversion efficiencies quite well correlates with the I_SC_ values. The CZTSSe solar cells made from the films sulfurized at 450 °C and 420 °C has the highest conversion efficiencies compared to those sulfurized at 380 °C degrees or as-deposited films. IPCE of the higher temperature sulfurized films is particularly increased in the longer wavelength region. This suggest that film sulfurization at 420 and 450 °C degrees provides better material quality required for solar cell devices because of the improved charge extraction and collection efficiencies in the whole spectral range. We also plotted the integrated current density from the IPCE spectra which showed quite good correlation with the J_SC_ values obtained from *I-V* measurements.

**Table 5:** Summary of optoelectronic parameters of CZTSSe based solar cells.

Sample	*V*_*OC*_, mV	*J*, mA/cm^2^	FF, %	PCE, %
As-deposited	255	24.69	36.6	2.73
380 °C	287	22.57	37.5	3.14
420 °C	300	25.17	39.4	3.48
450 °C	280	27.46	34.7	2.97

## Conclusions

This study reports the effects of the sulfurization temperature of CZTS films on the quality of CZTSSe absorbing material and the performance of CZTSSe-based solar cells. We found that the additional low temperature sulfurization of CZTS films is an inevitable process to produce high quality CZTSSe absorber materials. The choice of sulfurization temperature is a particularly important factor that can greatly affect the final quality of the CZTSSe films and the performance of the solar cells. The employed XRD diffractometer equipped with monochromatic Cu Kα1 radiation source and Raman spectroscopy evidenced that the highest quality CZTSSe material i.e. containing largest crystal sizes, a reduced defect densities and the lowest structural disorder for the CZTS films pre-sulfurized at 420 °C. This study also highlights the importance of using Sb_2_Se_3_ as a buffer layer for the fabrication of CZTSSe solar cells with reproducible performance parameters. The CZTSSe-based solar cells fabricated from sulfurized CZTS films at 420 °C exhibited a PCE of up to 3.48%, with an average efficiency of 3.1% and a standard deviation of 0.38%. This work shows that the optimization of the sulfurization conditions and appropriate chose of the buffer layer are important factors to further improve quality and performance of the spray pyrolysis deposited CZTSSe solar cells.

## Supplementary Information

Supplementary Information.

## Data Availability

The data that support the fndings of this study are available from the corresponding author upon reasonable request.
