# Supplementary material for: Improvement of CZTSSe film quality and superstrate solar cell performance through optimized post-deposition annealing
Source: Sci Rep. 2022 Sep 28;12:16170. doi: 10.1038/s41598-022-20670-1 (PMC9519875; doi:10.1038/s41598-022-20670-1)
Supplement: Supplementary file 1 — Supplementary Information. [file 41598_2022_20670_MOESM1_ESM.docx]

**Improvement of CZTSSe Film Quality and Superstrate Solar Cell Performance Through Optimized Post-Deposition Annealing**

*V. Pakštas^1^, G. Grincienė^1^, A. Selskis^1^, S. Balakauskas ^1^, M. Talaikis^1, 2^, L. Bruc^3^, N. Curmei^3^, G. Niaura^1^, M. Franckevičius^1,*^*

^1^ Center for Physical Sciences and Technology, Sauletekio av. 3, LT-10257 Vilnius, Lithuania

^2^ Institute of Biochemistry, Life Sciences Center, Vilnius University, Sauletekio 7, LT-10257, Lithuania

^3^ Institute of Applied Physics, 5 Academiei str. Chisinau, MD-2028, Moldova

**Figure S1**. Raman spectra of CZTS as-deposited and later annealed at different temperatures. The spectral range of 220–470 cm^-1^ was fitted using Gaussian-Lorentzian shape components. Spectra were recorded with 785 nm excitation wavelength.

**Table T1**. Chemical composition of the CZTS films.

| Sample |  | Cu, at. % | Zn, at. % | Sn, at. % | S, at. % | Cl, at. % |
| --- | --- | --- | --- | --- | --- | --- |
| As-deposited | V10 | 22,44 | 13,21 | 12,67 | 49,71 | 1,88 |
| 380° C | V1-5 | 22,54 | 13,85 | 12,82 | 49,47 | 1,12 |
| 420° C | V1-8 | 22,67 | 14,28 | 12,52 | 49,89 | 0,17 |
| 450° C | V1-12 | 22,32 | 14,18 | 12,94 | 50,48 | 0,08 |

**Figure S2.** Raman spectra of as-deposited CZTS films not subjected to the selenization. CZTS was deposited on FTO/TiO_2_ with Sb_2_Se_3_ layer. Raman spectra measured from the top side of CZTS (orange) and from the bottom (green).


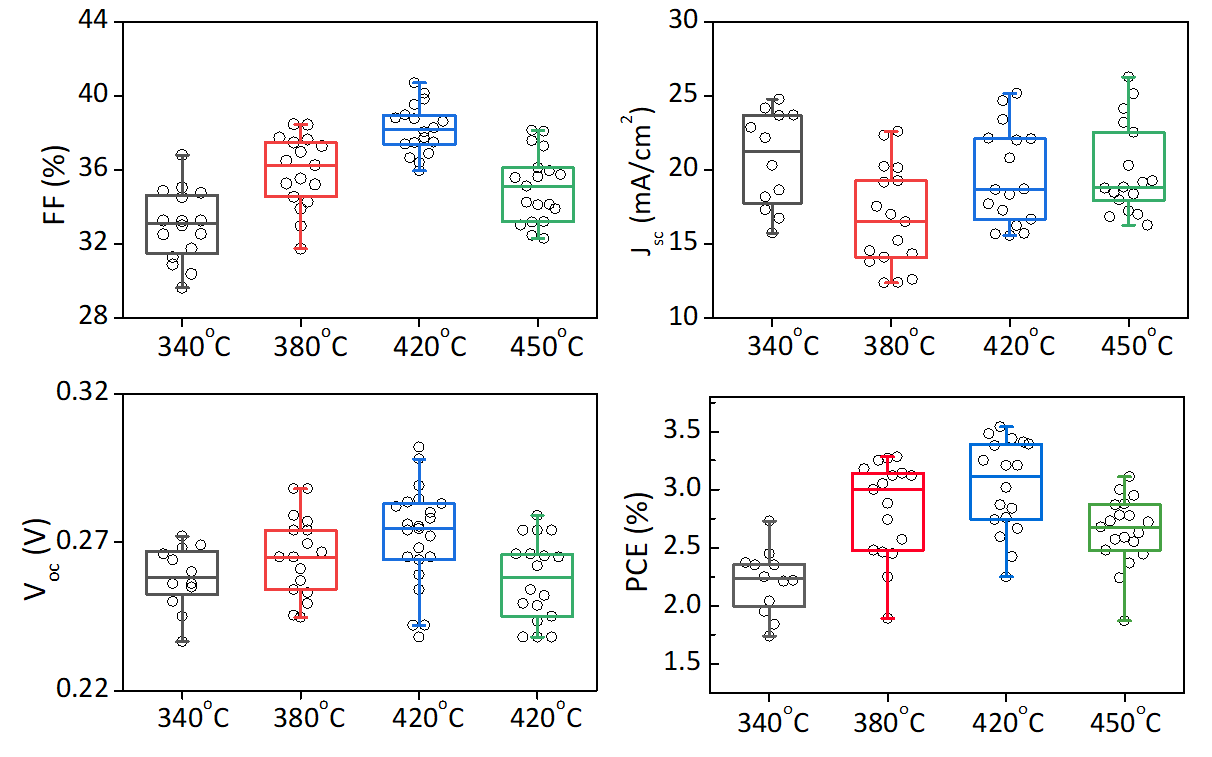


**Figure S3**. Statistical distribution of photovoltaic parameters of CZTSSe solar cells from batches of more than 10 devices.

Figure S4. IPCE spectra and integrated IPCE of the CZTSSe solar cells.
